# Enhancing Communication in Minimally Verbal Autistic Children: A Study on NAO-Assisted Therapy

**DOI:** 10.3390/jcm14113735

**Published:** 2025-05-26

**Authors:** Marcella Di Cara, Margherita La Fauci, Maria Tresoldi, Maria Rita Caputo, Daniele Borzelli, Roberta Maggio, Caterina Campestre, Antonella Barbera, Adriana Piccolo, Carmela De Domenico, Massimo Di Blasi, Rocco Salvatore Calabrò, Emanuela Tripodi, Caterina Impallomeni, Francesca Cucinotta

**Affiliations:** 1 IRCCS Centro Neurolesi Bonino Pulejo, Via Palermo, Contrada Casazza, 98124 Messina, Italy; marcella.dicara@irccsme.it (M.D.C.); maria.tresoldi@polime.it (M.T.); mariarita.caputo@irccsme.it (M.R.C.); adriana.piccolo@irccsme.it (A.P.); carmela.dedomenico@irccsme.it (C.D.D.); massimo.diblasi@irccsme.it (M.D.B.); roccos.calabro@irccsme.it (R.S.C.); emanuela.tripodi@irccsme.it (E.T.); caterina.impallomeni@irccsme.it (C.I.); francesca.cucinotta@irccsme.it (F.C.); 2 Otorhinolaryngology and Auditory Microsurgery Unit, Department of Experimental Medical-Surgery, Specialist and Odontostomatologica Science, University of Messina, 98122 Messina, Italy; 3 Department of Biomedical and Dental Sciences and Morphofunctional Imaging, University of Messina, 98122 Messina, Italy; daniele.borzelli@unime.it; 4 Laboratory of Neuromotor Physiology, IRCCS Santa Lucia Foundation, 00179 Rome, Italy; 5 Progetto Dopo di Noi Societa Cooperativa Arl, Via Kennedy 217, 98051 Barcellona Pozzo di Gotto Messina, Italy; maggioroberta19@gmail.com (R.M.); katiacampestre@libero.it (C.C.); antonella.barbera@me.com (A.B.)

**Keywords:** minimally verbal, autism spectrum disorder, NAO robot, social robotics, rehabilitation, communication initiative

## Abstract

**Background/Objectives**: Minimally verbal autistic children face significant communication challenges, often unmet by traditional therapies. Social robots, like NAO, offer predictable, structured interactions that may improve engagement and language skills. This study aimed to evaluate the effectiveness of NAO-assisted therapy in improving communication and social interaction in minimally verbal autistic children compared to standard therapeutic approaches. **Methods**: In a single-blind, randomized, controlled study, 37 autistic children aged 4–12 years were assigned to either NAO-assisted therapy or standard speech therapy. Participants were assigned to either an NAO-assisted therapy group or a standard speech therapy control group. The intervention included 12 weekly 45 min sessions. Communication outcomes were measured using the Language Development Level Test (TVL) and mand request observations. **Results**: All 37 participants completed the 12 sessions without adverse events, highlighting the intervention’s feasibility and safety. Children in the NAO-assisted therapy group showed greater improvements in verbal communication (on average, 159 ± 49% more children exhibited improvement across verbal aspects (range: 107–284%; *p* < 0.001)) particularly in spontaneous communication, compared to the control group. The therapy also increased mand production (from 6.8 ± 4.3 in session 1 to 16.7 ± 7.7 in session 12; *p* < 0.001; average gain: 0.9 per session), demonstrating steady growth in communicative initiative. These findings underscore the structured and engaging nature of NAO-assisted therapy in supporting consistent progress in communication skills. **Conclusions**: NAO-assisted therapy is a promising, safe, and effective intervention for enhancing communication in minimally verbal autistic children, offering unique benefits in promoting spontaneous and consistent verbal engagement.

## 1. Introduction

Communication is an essential aspect of human interaction, yet for approximately 25–30% of children with autism spectrum disorder (ASD), a neurodevelopmental disorder as classified by the DSM-5, it remains a significant barrier [1,2]. ASD is a complex neurodevelopmental condition characterized by deficits in social communication and restricted, repetitive behavior patterns, interests, or activities [3]. A recent review defines “minimally verbal” (MV) children as those who produce fewer than 20 functional and intelligible words or phrases in natural communication contexts. These children often exhibit significant limitations in phonetic and speech imitation abilities and lack productive syntax, restricting their capacity for meaningful verbal interactions [4]. MV autistic children represent a particularly heterogeneous population. Recent research identified distinct subgroups based on varying profiles of cognitive, linguistic, and social communication abilities [5]. This heterogeneity presents unique challenges for developing interventions, as children’s responses to therapy can vary significantly depending on their strengths and limitations [6]. Interventions must, therefore, be adaptable and targeted to address these diverse needs effectively, as recent research shows that response rates to rehabilitative therapies can differ significantly depending on the therapeutic approach used [7]. Traditional treatments, such as speech therapy, applied behavior analysis (ABA), and augmentative and alternative communication (AAC) systems, have demonstrated some efficacy in improving communication skills [1,8,9]. However, their success is often limited by the need for substantial customization and the child’s varying levels of engagement and responsiveness [10,11,12]. These limitations underscore the need for innovative interventions that are both adaptable and engaging. In this context, virtual reality (VR) and social robotics have emerged as promising tools in speech and language interventions [13,14]. VR has demonstrated enhanced engagement and improved communication outcomes in children with developmental language disorders [15], while social robotics, including humanoid robots, like the NAO, offer interactive features, such as speech recognition, gestures, and responses to touch and movement, making them particularly suited for engaging MV autistic children [16,17,18]. Previous studies on NAO-assisted therapy have shown encouraging results, including increased verbal initiation, improved social interaction, and reduced anxiety in autistic children [19,20,21]. Unlike conventional therapies, NAO provides consistent, predictable feedback and interactive tasks tailored to each child’s needs, such as prompting verbalizations through structured questioning or gestural cues [22,23,24]. The success of these initiatives is frequently constrained by two factors. Firstly, a significant degree of customization is required, and, secondly, the child’s level of engagement and responsiveness is subject to variation [25,26,27].

This study evaluates whether integrating the NAO humanoid robot into a rehabilitation program can improve communicative initiative and social interaction in MV autistic children. The objective is to determine whether NAO-assisted therapy results in greater improvements in verbal communication and social engagement when compared to standard therapeutic approaches.

By systematically comparing NAO-assisted interventions with as-usual therapeutic approaches, the study seeks to provide evidence on the feasibility and efficacy of robotic therapies, with the ultimate goal of enhancing clinical practices and improving the quality of life for MV children and their families.

## 2. Materials and Methods

### 2.1. Study Design

A single-blind, randomized, controlled study was performed at the Child Neuropsychiatry service of the IRCCS Centro Neurolesi “Bonino Pulejo” in Messina, Italy, specifically within outpatient clinics dedicated to neurodevelopmental disorders. This study was conducted in accordance with the Declaration of Helsinki [28] and received approval from the Ethical Committee IRCCS Sicilia Centro Neurolesi “Bonino-Pulejo” (n. 15/19). This clinical trial adheres to CONSORT guidelines [29] and has been registered at http://www.clinicaltrials.com (accessed on 28 January 2025) (NCT06805916). The trial was retrospectively registered as participant enrollment began before registration was finalized. The study protocol remained consistent with the version approved by the ethics committee. Participants were identified through referrals from clinicians at the neuropsychiatry service, and consent was obtained in face-to-face meetings with parents or guardians after a thorough explanation of the study objectives and procedures. In accordance with ethical standards and institutional policy, children between the ages of 6 and 11 were also provided with an age-appropriate assent form, and their verbal and/or written assent was obtained before participation. Participants were randomly assigned to the experimental treatment group (which received the NAO-assisted speech therapy intervention) or the control group (which received as-usual speech therapy approaches, without the robot) using a computer-generated randomization sequence. The NAO-assisted therapy was delivered on a one-on-one basis in a dedicated therapy room within the outpatient neurodevelopmental clinic. Sessions were conducted by certified professionals trained both in NAO-assisted intervention and in the treatment of autistic children, specifically two licensed speech therapists and two psychologists. The randomization process utilized a block randomization technique with varying block sizes to ensure balanced group allocation. A software program generated the randomization sequence, and assignments were securely stored in a centralized database accessible only to the research coordinator. This ensured allocation concealment and minimized potential biases. This trial followed a parallel design with a 1:1 allocation ratio, where participants were equally randomized to either the experimental treatment group or the control group. The intervention was delivered according to a standardized protocol to ensure consistency across sessions and therapists. All clinicians received joint training and followed shared session plans and therapeutic objectives. The procedural consistency was supported through structured session plans, shared training, and regular team meetings among the therapists to ensure adherence to the intervention protocol. No changes to the study methods, including eligibility criteria or intervention protocols, were made after the commencement of the trial.

### 2.2. Inclusion Criteria

This study involved 40 children diagnosed with ASD by experienced psychiatrists according to DSM-5 criteria [30]. The sample size was determined to balance the exploratory nature of the study, focusing on feasibility, safety, and preliminary efficacy, with the available resources and the need for manageable recruitment and retention processes. To support the clinical diagnosis, standardized tools were employed, such as Autism Diagnostic Observation Schedule—Second Edition (ADOS-2) [31] for diagnostic observation and the Vineland Adaptive Behavior Scales II (VABS-II) [32] for assessing adaptive skills. Cognitive abilities were evaluated using the Leiter-3 scale [33], and verbal expression and comprehension skills assessments were conducted to identify the clinical characteristics of the MV profile.

Eligibility criteria included (1) a diagnosis of ASD according to DSM-5; (2) being within the age range of 4–12 years; (3) a minimally verbal profile; (4) the absence of severe comorbidities, such as uncontrolled epilepsy or sensory impairments; (5) consistent attendance in the therapy program for the total number of sessions scheduled; and (6) signed informed consent.

The exclusion criteria were as follows: (a) severe motor or sensory impairments, such as significant cerebral palsy or blindness, that could interfere with robotic activities; (b) severe neurological disorders or genetic syndromes; (c) acute or chronic medical conditions that might compromise safe participation; and (d) severe maladaptive behaviors, including persistent aggressive behaviors or self-injury, that could render therapy sessions unsafe.

### 2.3. Measures

Changes between pre-intervention (T0) and post-intervention (T1) were assessed by independent evaluators who were blinded to the treatment assignments. No changes to the trial assessments or measurements were made after the trial commenced. Each participant underwent evaluations both before and after the intervention, conducted by the same assessor to ensure consistency. Feasibility was analyzed based on service utilization metrics, including participant adherence rates to the rehabilitation programs. Adherence to the intervention was monitored by recording attendance at each session. Safety was evaluated by observing participants’ responses to the robot during the intervention sessions. Particular attention was given to signs of discomfort, fear, or other adverse reactions, which were recorded systematically. Additionally, therapists were instructed to document any incidents or behavioral concerns that could indicate a negative reaction to the NAO robot. The preliminary efficacy of the experimental intervention was assessed using two primary outcome measures. The primary measure of communicative ability was the Language Development Level Test (TVL) [34], a standardized assessment that evaluates different aspects of verbal communication. The following subscales of the TVL were used:

− Construction spontaneous production (Cons. Spont): The number of words spontaneously produced by the child during the session.
− Comprehension of words (CW): The ability of the child to understand and respond to spoken words.
− Comprehension of sentences (CS): The ability of the child to comprehend and respond to spoken sentences.
− Comprehension total (Comp. total): The cumulative score derived from CW and CS, representing overall language comprehension.
− Nominating body parts (NB) and objects (NO): The child’s ability to name common objects and body parts.
− Naming total: A combined measure of NB and NO, reflecting the child’s ability to label objects and body parts.
− Phonological and morphosyntactic accuracy (AP and AM): The accuracy with which the child produces sounds and follows grammatical structures in their speech.
− Sentence construction (SC): The ability of the child to construct grammatically correct sentences.
− Consistent period (Cons. Period): The duration of time during which the child consistently produces verbal responses.
− Mean length of the utterance (Cons. MLU): The average length of verbal expressions in morphemes, calculated to assess syntactic complexity.

In addition to the TVL, observational data were collected during each session to measure mand requests produced by children in the experimental group. Mand requests were classified as spontaneous if initiated independently by the child or prompted if initiated following a verbal or gestural cue from the therapist. Data were recorded using a standardized observational grid and analyzed to assess changes in communicative initiative. Inter-observer agreement (IOA) was assessed via a second observer, who independently recorded data during 50% of randomly selected baseline, treatment, maintenance, and generalization sessions. IOA was calculated using point-by-point agreement by dividing the number of agreements by the total number of agreements and disagreements, then multiplying by 100%. IOA was 100% across participants.

### 2.4. Intervention

The experimental treatment group participated in a structured rehabilitative program integrating the NAO humanoid robot. The NAO robot (SoftBank Robotics Group, Paris, France) is a programmable humanoid robot equipped with speech recognition, gesture recognition, and the ability to respond to touch and movement. The robot’s interactive capabilities are designed to engage children in communication and social behaviors, providing consistent and predictable interactions that are less overwhelming than human interactions.

The intervention consisted of 12 speech therapy sessions, conducted once a week over 12 weeks, in outpatient clinics dedicated to neurodevelopmental disorders. Each 45 min session was part of a structured speech therapy program, specifically designed to encourage children to engage in spontaneous verbalizations, express their needs, and interact socially. Follow-up assessments were conducted immediately after the completion of the intervention. The NAO robot was used as an assistive tool alongside the therapist to enhance engagement and communication outcomes. During each session, the robot performed a series of programmed tasks aimed at prompting communicative behaviors, such as asking questions, performing gestures, and reacting to the child’s actions. The NAO robot performed tasks, such as playing interactive games (e.g., identifying objects shown on a screen), asking open-ended questions (e.g., “What is your favorite color?”), and mimicking gestures or sounds to elicit responses from the child. The therapist provided support, guided interactions, and reinforced appropriate verbal responses. In addition to the robot, each session incorporated complementary materials, such as printed worksheets and visual aids, tailored to support the session’s communicative goals. These materials were used to reinforce target vocabulary, support task comprehension, and facilitate interaction. The NAO robot served as a facilitator to enhance the child’s engagement and promote communicative exchanges within a structured therapeutic context. The control group received as-usual speech therapy for an equivalent amount of time. The objectives of the control group’s intervention were aligned with those of the experimental group, focusing on encouraging spontaneous verbalizations, enabling the expression of needs, and promoting social interaction. These treatments were delivered by trained therapists according to established protocols for MV autistic children.

### 2.5. Statistics

Statistical analyses were performed using MATLAB’s (version 2023b) built-in functions.

The effect of the NAO humanoid robot was assessed by evaluating the improvement in different aspects of verbal communication between T0 and T1. Specifically, for each verbal aspect, we calculated the proportion of children who showed improvement between T0 and T1 in both the NAO-assisted therapy group and the classical therapy group. The effect of the NAO was then assessed using the Wilcoxon signed-rank test (MATLAB function *signrank*), a non-parametric, non-paired test.

The progression of mand across sessions was also analyzed in children who practiced with the NAO robot. This was assessed using a linear mixed model, implemented with the following equation:

$$mand\sim sessionId+\left(1|patientId\right)$$

where *sessionId* represented the sessions and was treated as a fixed effect (modeled as a continuous variable) while *patientId* indicated each patient and was modeled as a random effect. The linear mixed model was implemented in MATLAB using the fitlme function.

The relationship between verbal aspects and the increase in mand across sessions was also investigated. For each participant, the slope of mand was computed using linear regression (MATLAB function regress). Each verbal aspect, measured for children undergoing NAO-assisted rehabilitation, was assigned a binomial value. Separate linear models were then built to assess the effect of each verbal aspect, both at T0 and T1, on the slope of mand, using the following equation:

$${mand}_{slope}\sim {verbal}_{T1}+{verbal}_{T0}$$

where ${mand}_{slope}$ represents the rate of change in mand across sessions, while ${verbal}_{T0}$ and ${verbal}_{T1}$ represent the respective verbal aspects measured at T0 and T1.

Finally, we examined whether the measures obtained from the ADOS-2, VABS-II, and Leiter-3 scales influenced the verbal aspects. The improvement in a given verbal aspect was defined as a binomial variable, set to 1 if the aspect increased from T0 to T1 and 0 otherwise. The type of rehabilitation (standard or NAO-assisted) was modeled as a categorical binomial variable, while the ADOS-2, VABS-II, or Leiter-3 scores were treated as continuous variables. Additionally, we evaluated the interaction effect between the type of rehabilitation and the ADOS-2, VABS-II, or Leiter-3 scores.

For all statistical analyses, *p*-values < 0.05 were considered statistically significant.

## 3. Results

### 3.1. Participant Recruitment and Completion

**Figure 1:**
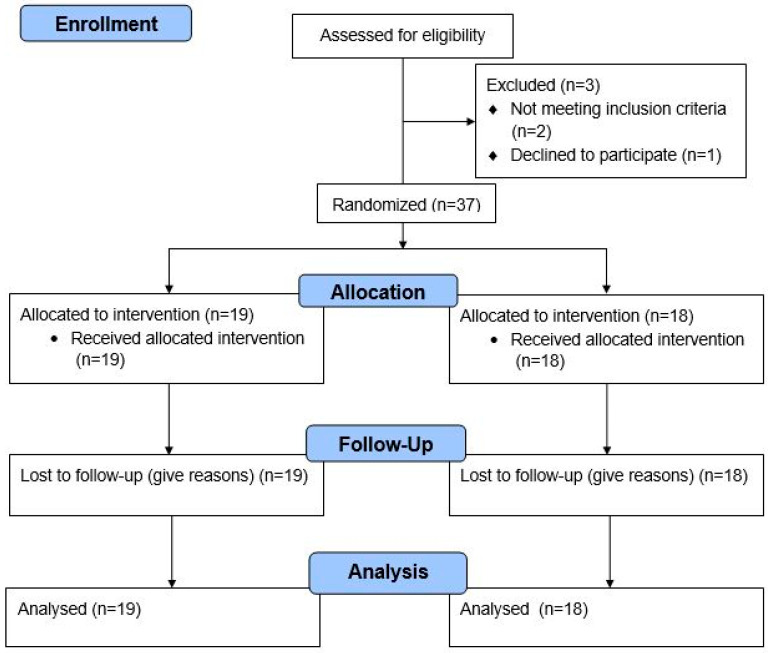
The CONSORT flowchart with detailed information about participants in the study.

A total of 40 participants were assessed for eligibility. Of these, 2 did not meet the inclusion criteria and were excluded from the study. Among the 38 participants who met the eligibility criteria, one participant, assigned to the control group, withdrew before the intervention due to personal reasons. Detailed information on patients is reported in the CONSORT flowchart of the study in Figure 1. The final sample consisted of 37 participants. A total of 18 participants (15 males and 3 females; mean age 7.3 ± 1.7 years) were assigned to the control group, who received as-usual speech treatment, while 19 participants (16 males and 3 females; mean age 8.1 ± 2.5 years) were assigned to the experimental group, who underwent speech therapy with NAO. A more detailed description of the two groups is provided in Table 1. No significant difference was detected in the age (*p* = 0.427, Wilcoxon non-parametric rank sum test) between the experimental and control groups. Similarly, no significant differences were detected (Wilcoxon non-parametric rank sum test) between (a) cognitive levels, assessed using the Leiter-3 (*p* = 0.199); (b) adaptive behavior, evaluated using the VABS (*p* = 0.819); (c) autism severity level, assessed using Ados total score (*p* = 0.436); and (d) comparison score (*p* = 0.741) between the experimental and control groups. Participants were recruited between April 2023 and April 2024.

### 3.2. Feasibility and Safety

All 37 children presented in this study completed all 12 sessions, resulting in a 100% retention rate for both groups. No adverse events, negative reactions, or signs of discomfort were observed in any participant during the intervention sessions. The safety of the intervention was carefully monitored throughout the study, with therapists systematically documenting participants’ behaviors and responses to the robot-assisted therapy. All participants tolerated the intervention, and no incidents were reported that would raise safety concerns.

### 3.3. The NAO-Assisted Rehabilitation Showed Better Outcomes

**Figure 2:**
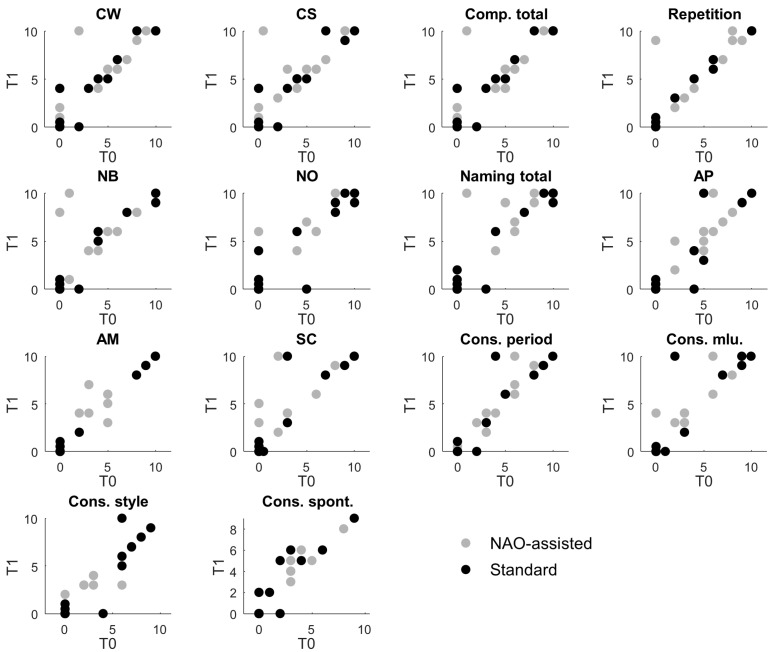
Measured verbal aspects. Each panel represents a different verbal aspect. Each dot corresponds to the score of an individual participant for that specific aspect at T1 (y-axis) versus T0 (x-axis). Patients who underwent NAO-assisted rehabilitation are shown as gray dots, while those who received standard therapy are shown as black dots. CW: comprehension of words; CS: comprehension of sentences; Comp. total: comprehension total; NB: nominating body parts; NO: nominating object; AP: phonological accuracy; AM: morphosyntactic accuracy; SC: sentence construction; Cons. Period: construction period; Cons. Mlu: construction mean length of utterance; Cons. Style: construction style; Cons. Spont: construction spontaneous production.

For the primary objective (e.g., evaluating the improvement in communication skills using the TVL), all participants were included in the analysis (Figure 2). No data were missing, and all participants completed the assessments at T0 and T1.

**Figure 3:**
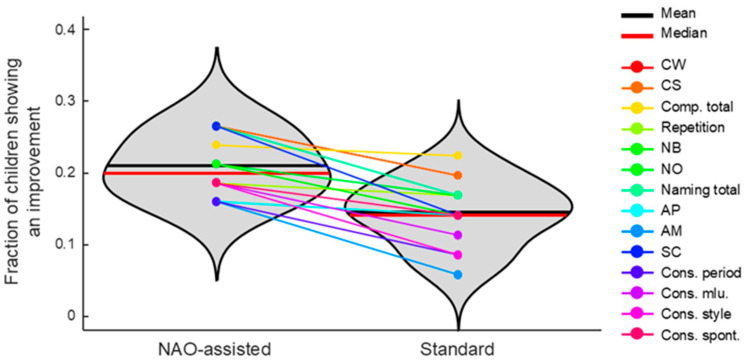
Fraction of children who underwent rehabilitation with the NAO robot (left) or standard rehabilitation (right) and showed an increase in verbal aspects (color-coded). The thick black and red lines represent the mean and median values, respectively, across all children in each rehabilitation group. CW: comprehension of words; CS: comprehension of sentences; Comp. total: comprehension total; NB: nominating body parts; NO: nominating object; AP: phonological accuracy; AM: morphosyntactic accuracy; SC: sentence construction; Cons. Period: construction period; Cons. Mlu: construction mean length of utterance; Cons. Style: construction style; Cons. Spont: construction spontaneous production.

The fraction of children showing an improvement in each aspect of verbal communication between T0 and T1 was compared between those who practiced with the NAO robot and those who underwent classical rehabilitation (see Figure 3). On average, across all verbal aspects, rehabilitation with the NAO robot resulted in 159 ± 49% (mean ± SD across verbal aspects) more children exhibiting an improvement compared to those receiving classical rehabilitation, with increases ranging from 107% (Comp_Total) to 284% (AM).

A Wilcoxon signed-rank test conducted on the fraction of children who showed an improvement in verbal communication revealed significantly higher values in those who practiced rehabilitation with NAO (*p* < 0.001).

### 3.4. The Mand Increased Across Sessions

Mand requests were evaluated exclusively in the experimental group because this measure was directly linked to the specific objectives of the robot-assisted therapy. The NAO robot was programmed to prompt communicative behaviors through interactive activities, making mand requests a relevant indicator of the intervention’s impact. Conversely, the control group received conventional therapy, which did not include comparable structured activities specifically aimed at eliciting mand responses.

**Figure 4:**
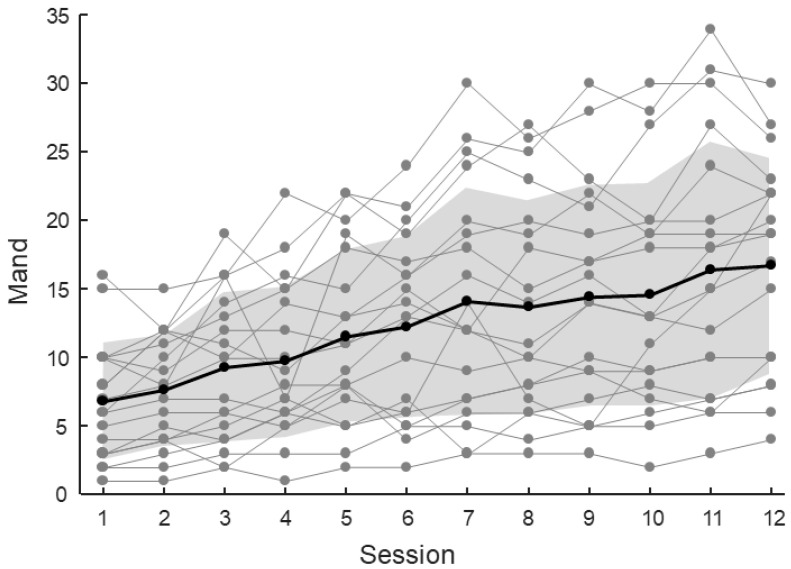
Evolution of the mand across sessions. Gray lines indicate individual patient values, while the black line represents the mean value. Shaded areas correspond to mean ± SD across patients.

The mand (Figure 4) increased from session 1 (6.8 ± 4.3, mean ± SD across patients) to session 12 (16.7 ± 7.7, mean ± SD across patients). The progression of the mand across sessions with the NAO robot was analyzed using a linear mixed model. The analysis indicated a significant linear increase in the mand over sessions (*p* < 0.001), with an average growth of 0.9 per session.

### 3.5. Influence of Verbal Communication Aspects on the Mand

We then investigated which verbal communication aspects at T0 and T1 influenced the growth of the mand, measured as the linear regression slope across sessions.

The linear model analysis revealed that only CW at T1 exhibited a significant positive association with the mand slope (*p* = 0.022). Thus, greater comprehension of words at T1 was associated with higher mand growth. Conversely, NO at both T0 and T1 had significant effects on the mand slope. However, while NO at T1 was positively associated with the mand slope (*p* = 0.038), NO at T0 was negatively associated (*p* = 0.038). This suggests that higher naming object capabilities at T1 were linked to greater mand growth, whereas higher naming object skills at T0 were associated with lower mand growth, possibly due to a reduced potential for improvement in this aspect.

All other verbal aspects, whether measured at T0 or T1, showed no significant effect on the mand (*p* > 0.066). 

## 4. Discussion

### 4.1. Relevance to Previous Research

Minimally verbal communication is a frequent characteristic in children with neurodevelopmental disorders, particularly in ASD [35]. This limited communication characteristic significantly impacts social interactions, learning opportunities, and overall quality of life [36]. Consequently, targeted rehabilitative interventions are crucial to fostering language acquisition and improving communication abilities in these individuals. Robot-assisted therapy has emerged as a promising approach to addressing the challenges of MV children, leveraging structured, predictable, and interactive frameworks that can enhance engagement and learning [37]. Several studies have demonstrated that social robots can improve joint attention, turn-taking, and speech production [16,38]. One of the key strengths of the NAO-assisted approach is its ability to sustain engagement and motivation, factors that are crucial in language acquisition. The present study contributes to this body of knowledge by examining the effects of NAO-assisted rehabilitation on verbal development, particularly in comparison to traditional therapy approaches. A key strength of this study is that the experimental and control groups were well-balanced in terms of age, cognitive levels (Leiter-3), adaptive behavior (VABS), and ASD severity (ADOS scores). This ensures that the observed improvements in communication were likely due to the NAO-assisted therapy rather than pre-existing differences between groups.

### 4.2. Contributions of the Present Study

Building upon previous findings, our study not only corroborates the potential of NAO-assisted therapy but also expands the current understanding by providing a more comprehensive evaluation of verbal development and communicative initiative. While previous studies utilizing the NAO robot in ASD therapy focused on joint attention with motion tracking and prompt-response measures [39,40], and assessed social behaviors and stereotyped movements using the GARS-2 scale [41,42], our study differs by not only employing a larger sample size but also by evaluating verbal development and communicative initiative through a combination of TVL and mand measures. This ensures a more comprehensive assessment of communication progress and highlights the unique contribution of our NAO-assisted intervention.

Additionally, the intervention demonstrated high feasibility and safety, as evidenced by the 100% retention rate and the absence of any adverse events, negative reactions, or discomfort during sessions. The structured nature of the therapy allowed systematic monitoring, and all participants tolerated the intervention well. This reinforces the potential of NAO-assisted therapy as a reliable and well-tolerated therapeutic approach, which could be implemented in clinical and educational settings. The results indicate that NAO-assisted rehabilitation can provide significant improvements in verbal intentionality in MV children. One key finding confirms that the experimental treatment significantly enhanced communication abilities, suggesting that robotic-assisted therapy may facilitate progress in language acquisition at a faster rate compared to as-usual interventions. Prior research has indicated that structured, technology-driven interventions can reinforce verbal expression in ASD populations [43]. The structured and interactive nature of the NAO-assisted intervention likely contributed to maintaining engagement, a crucial factor in sustaining communication progress [44]. These findings reinforce the potential of robotic-assisted therapy as an effective complement to traditional speech and language interventions. However, while our results highlight the promise of NAO-assisted therapy, future studies should explore the specific mechanisms underlying this effect, such as the predictability and social responsiveness of the robot, to determine which factors contribute most to these positive outcomes. A second major finding concerns the increase in communicative initiative, as measured by mand production. Unlike previous studies reporting non-linear and inconsistent gains in spontaneous communication for MV children [45,46], our data show a clear and consistent linear increase in mand production over time. This finding suggests that NAO-assisted therapy provides an environment conducive to both initiating and sustaining communicative interactions. Traditional interventions often struggle to maintain steady progress in spontaneous communication, whereas robotic-assisted therapy appears to continuously reinforce and strengthen such behaviors over multiple sessions [47]. Given that developing spontaneous communicative behaviors remains one of the most challenging aspects of language interventions for ASD, our findings underscore the potential of robotic-assisted therapy in promoting independent communication. However, further studies are needed to assess whether these improvements generalize to everyday settings and remain stable over time, as noted in the study limitations. Additionally, a third critical result of this study is the relationship between linguistic improvements and increases in communicative initiative. Specifically, improvements in word comprehension and object naming at T1 were significantly associated with a steeper increase in mand production. These findings support previous research suggesting that stronger lexical and semantic processing abilities are linked to increased expressive language use and spontaneous communicative attempts [48,49]. Interestingly, we also found that higher scores in naming objects at T0 were associated with a lower rate of mand increase. This inverse relationship may reflect a ceiling effect, where children who already had strong object-naming abilities at baseline exhibited less room for further improvement in their communicative initiative. Similar trends have been reported in language intervention studies, where children with higher baseline verbal skills demonstrate more stable but slower progress compared to those with greater initial deficits [50,51]. These findings emphasize the importance of tailoring intervention strategies based on initial linguistic abilities, ensuring that children with stronger pre-existing verbal skills receive appropriately advanced communicative support.

### 4.3. Implications for Practice and Integration

One potential direction for practical implementation involves enabling autonomous interaction with the NAO robot under non-clinician supervision, such as that of parents or tutors. If children can engage with the robot under parental or tutor supervision, this could significantly extend therapy time beyond scheduled sessions, increasing exposure to structured communication exercises. Prior research has suggested that increased therapy time correlates with greater language gains in ASD interventions [52]. Future research should investigate whether unsupervised, parent-guided interactions with NAO yield comparable results to therapist-led sessions, and how hybrid models of robotic and human intervention could be optimized for maximal benefit. Furthermore, the integration of robotic-assisted therapy into clinical and educational settings remains a crucial area of future development. While our study demonstrated the efficacy of NAO in structured experimental conditions, real-world implementation presents several challenges, including cost, accessibility, and the need for therapist and caregiver training. Future research should explore strategies to facilitate widespread adoption, ensuring that NAO-assisted therapy is both scalable and sustainable in diverse therapeutic contexts.

### 4.4. Future Directions

While our results highlight the promise of NAO-assisted therapy, future studies should explore the specific mechanisms underlying this effect, such as the predictability and social responsiveness of the robot, to determine which factors contribute most to these positive outcomes. Moreover, future investigations should explore whether specific features of NAO-assisted therapy, such as reinforcement mechanisms, multimodal feedback, or interactive turn-taking, play a primary role in fostering communication gains. Identifying these components would allow for further optimization of robotic-assisted interventions to maximize their impact on speech and language rehabilitation.

Although not included in the present study, future research should also incorporate social validity assessments, such as parental feedback or satisfaction surveys, to better evaluate the acceptability, perceived benefit, and real-world value of the intervention from the caregiver’s perspective.

## 5. Conclusions

This study provides compelling evidence that NAO-assisted rehabilitation is an effective tool for improving both verbal communication and communicative initiative in minimally verbal children. The substantial improvements observed in verbal expression and functional communication exceed those typically reported in traditional therapy settings, highlighting the transformative potential of robotic interventions in language rehabilitation. Our findings align with recent discussions in autism intervention research, particularly the need for novel, engaging approaches, as highlighted by Vivanti [52]. Conventional therapies have produced mixed outcomes, emphasizing the necessity for interventions that are adaptable to the diverse needs of children with ASD. The NAO-assisted therapy addresses this gap by providing a structured yet interactive environment that enhances engagement, a key predictor of intervention success. This aligns with current recommendations advocating for the integration of technology-based methodologies in early intervention to improve both communicative outcomes and therapeutic engagement.

## 6. Limitations

Despite its promising findings, this study has several limitations that should be considered. First, the sample size was relatively small, which may limit the generalizability of the results. Future studies should aim to replicate these findings in larger and more diverse populations to confirm the efficacy of NAO-assisted therapy. Future studies should aim to replicate these findings in larger and more diverse populations to confirm the efficacy of NAO-assisted therapy. Second, the study focused only on short-term improvements, and it remains unclear whether the observed gains in communication abilities persist over time. Longitudinal studies should be conducted to assess the long-term sustainability of the intervention and whether these improvements generalize to real-world communication settings. Moreover, while our study identified key linguistic predictors of communicative initiative, additional research is needed to determine which aspects of robotic therapy contribute most to its success.

Finally, exploring hybrid intervention models, where robotic therapy is combined with conventional speech-language therapy, may provide the most effective strategy for enhancing verbal skills and communicative initiative in ASD.

## Figures and Tables

**Table 1:** Demographic characteristics of the participants.

	Experimental Group	Control Group	Total
Final Sample (n)	19	18	37
Females (n, %)	3 (15.8%)	3 (16.7%)	6 (16.2%)
Males (n, %)	16 (84.2%)	15 (83.3%)	31 (83.8%)
Age (years, mean ± SD)	8.1 ± 2.5	7.3 ± 1.7	7.7 ± 2.2
Leiter	73.3 ± 14.8	74.6 ± 14.9	73.9 ± 14.6
VABS	67.5 ± 20.3	71.0 ± 14.2	69.2 ± 17.5
Ados total scors	13.4 ± 5.3	14.6 ± 5.5	14.0 ± 5.4
Comparison score	6.5 ± 1.6	6.8 ± 1.9	6.6 ± 1.8

## Data Availability

Data and materials related to this study are available upon reasonable request from the corresponding author.

## Abbreviations

The following abbreviations are used in this manuscript:

ASD	Autism spectrum disorder
MV	Minimally verbal
ABA	Applied behavior analysis
AAC	Augmentative and alternative communication
VR	Virtual reality
ADOS	Autism Diagnostic Observation and Schedule
VABS	Vineland Adaptive Behavior Scales
TVL	Language Development Level Test
Cons. Spont	Construction spontaneous production
CW	Comprehension of words
CS	Comprehension of sentences
Comp. total	Comprehension total
NB	Nominating body parts
NO	Nominating objects
AP	Phonological accuracy
AM	Morphosyntactis accuracy
SC	Sentence construction
Cons. Period	Consistent period
Cons. MLU	Mean length of utterance
IOA	Inter-observer agreement
